# 

**Published:** 1868-01

**Authors:** 


### CONTENTS.

ORIGINAL COMMUNICATIONS.

Movements of the Soft-Solids and Liquid of the Blood ........ 9
Anæsthesia ........ 16
Care and Treatment of the Six Year Old Molars ........ 23
Microscopy of the Teeth— *Continued* ........ 28

PROCEEDINGS OF SOCIETIES.

Discussions in the Western New York Dental Association, at its Fifth Annual Meeting, held in the City of Lockport ........ 31

EDITORIAL.

New Rubber ........ 41
New Volume ........ 42
New Orleans Dental College ........ 43
Improvement in Plate Teeth ........ 44
Hall's Journal of Health ........ 44
Mississippi Valley Dental Association ........ 45
Ohio Dental College Association ........ 45
Close of the College Session ........ 46
Death from Chloroform ........ 46
Bibliographical ........ 49
The Teeth ........ 50
Simpson's Rubber—Price Reduced ........ 50

